# A 3.06 μm Single-Photon Avalanche Diode Pixel with Embedded Metal Contact and Power Grid on Deep Trench Pixel Isolation for High-Resolution Photon Counting †

**DOI:** 10.3390/s23218906

**Published:** 2023-11-01

**Authors:** Jun Ogi, Fumiaki Sano, Tatsuya Nakata, Yoshiki Kubo, Wataru Onishi, Charith Koswaththage, Takeya Mochizuki, Yoshiaki Tashiro, Kazuki Hizu, Takafumi Takatsuka, Iori Watanabe, Fumihiko Koga, Tomoyuki Hirano, Yusuke Oike

**Affiliations:** Sony Semiconductor Solutions Corporation, Atsugi-shi 243-0014, Japan; fumiaki.sano@sony.com (F.S.); tatsuya.nakata@sony.com (T.N.); yoshiki.kubo@sony.com (Y.K.); wataru.onishi@sony.com (W.O.); charith.koswaththage@sony.com (C.K.); takeya.mochizuki@sony.com (T.M.); yoshiaki.tashiro@sony.com (Y.T.); kazuki.hizu@sony.com (K.H.); takafumi.takatsuka@sony.com (T.T.); iori.watanabe@sony.com (I.W.); fumihiko.koga@sony.com (F.K.); tomoyuki.hirano@sony.com (T.H.); yusuke.oike@sony.com (Y.O.)

**Keywords:** SPAD, high-resolution photon counting, two-step deep trench isolation, embedded metal wiring

## Abstract

In this study, a 3.06 μm pitch single-photon avalanche diode (SPAD) pixel with an embedded metal contact and power grid on two-step deep trench isolation in the pixel is presented. The embedded metal contact can suppress edge breakdown and reduce the dark count rate to 15.8 cps with the optimized potential design. The embedded metal for the contact is also used as an optical shield and a low crosstalk probability of 0.4% is achieved, while the photon detection efficiency is as high as 57%. In addition, the integration of a power grid and the polysilicon resistor on SPAD pixels can help to reduce the voltage drop in anode power supply and reduce the power consumption with SPAD multiplication, respectively, in a large SPAD pixel array for a high-resolution photon-counting image sensor.

## 1. Introduction

Single-photon avalanche diode (SPAD) pixels have been developed for time-of-flight (ToF) range image sensors [1,2,3,4,5]. In addition, photon-counting imaging has been proposed as a promising technology for image acquisition with noiseless readout and high dynamic range (HDR) [6,7,8,9]. A SPAD image sensor with full digital readout is well-matched with the photon-counting architecture for global shutter image capture with noiseless readout and HDR by shrinking the SPAD pixel size and stacking a logic chip with pixel-parallel Cu-Cu connections [10,11,12,13]. Although the photon-counting technology can be applied for security, industrial, and scientific applications, achieving higher resolution by shrinking the pixel size and increasing the array size to the same size as a conventional complementary metal–oxide–semiconductor (CMOS) image sensor is still a challenge. Recently, the SPAD pixel size has been reduced to 5 μm or less to improve the pile-up problem and resolution of the SPAD-based image sensors [14,15,16]. However, the dark count rate (DCR) and photon detection efficiency (PDE) are significantly worse in smaller SPAD pixels compared to pixels larger than 6 μm [12,17,18]. This is due to edge breakdown (EBD) in the high electric field region at the pixel edge or the small avalanche region. The most recent research can resolve this problem by achieving over 80% PDE and under 5 cps (counts per second) DCR with a 3.0 μm pitch SPAD pixel [19]. However, the crosstalk is higher compared to a 6 μm SPAD pixel [17] due to the small pitch. In addition, the power consumption and voltage drop with SPAD multiplication must be improved in high-resolution photon-counting image sensors with a large array of such small pixels. We present a SPAD pixel with a pitch of 3.06 μm using an embedded metal contact and power grid on two-step deep trench isolation [20,21]. This study describes the details of the structure, fabrication process, potential design, and measurement results of the SPAD pixel, which suppresses EBD, crosstalk, and voltage drop, while simultaneously maintaining PDE and DCR. In addition, by incorporating a polysilicon resistor on the SPAD pixel, we demonstrate a decrease in charge per event (CPE) with SPAD multiplication.

## 2. Method

### 2.1. Pixel Structure

Table 1 shows the comparison between proposed SPAD pixel designs/structures for decreasing pixel size. The well-sharing pixel has been used for conventionally larger SPAD pixels with a pixel pitch of 6 μm or greater [16]. However, if this concept is implemented in the smaller SPAD pixels, the distance between the anode and cathode contact is not enough to relax the electrical field in the horizontal direction along the pixel surface at the pixel edge. The high electrical field at the pixel edge causes EBD and increases the DCR. A deeper multiplication region is introduced to relax the electric field at the pixel edge [15]. However, the deeper ion implantation for the N-well increases the variation in the breakdown voltage because higher-energy ion implantation is needed, and it is difficult to control the implantation depth and ion distribution. A shared guard ring removes the contact and the P-well between the pixels to decrease the electric field on the pixel edge [16]. In this design, the contact to the P-well must be placed around the pixel array and not within the pixel array. This causes an increase in the resistance for the P-well especially in a large SPAD pixel array and it induces instability of the SPAD characteristics.

In this study, we propose a new pixel structure to reduce the electric field on the pixel edge by introducing the embedded metal contact. The embedded contact deepens the anode contact region and increases the vertical distance between the anode and cathode contacts. This structure can resolve the compromise between the characteristics that plagued previous works. We propose this idea as one of the solutions for the SPAD pixel size decreasing below 3 um pitch, despite the complexity of the fabrication process.

**Figure 1:**
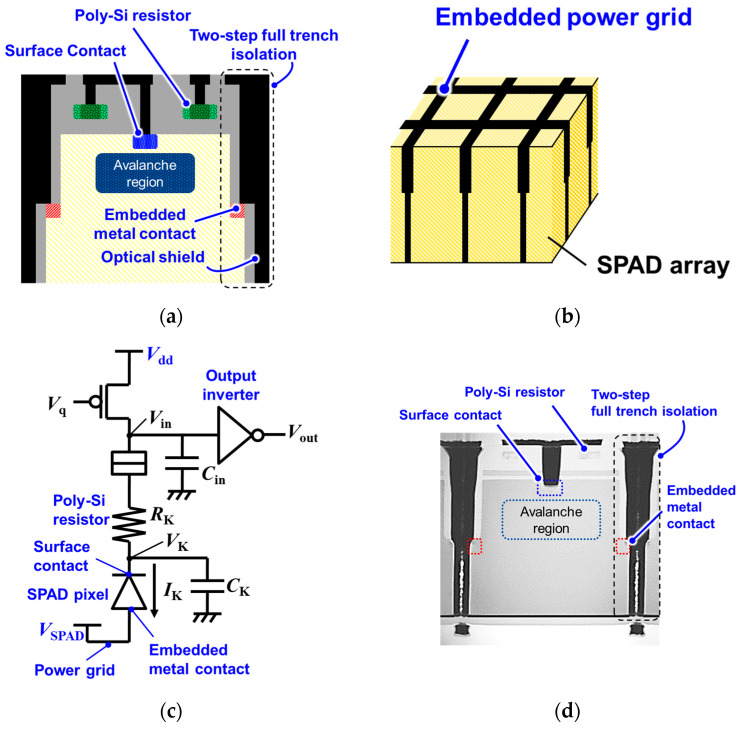
(**a**) Schematic diagram of a 3.06 μm SPAD pixel with an embedded metal contact on a two-step deep trench pixel isolation and (**b**) embedded power grid in a SPAD array. (**c**) Circuit diagram for a SPAD quenching circuit containing a polysilicon (Poly-Si) resistor. (**d**) Cross-sectional TEM image of the 3.06 μm SPAD pixel.

Figure 1a shows a schematic diagram of the developed 3.06-μm-pitch SPAD pixel with the embedded metal contact. One of the contacts in the pixel is embedded, with the embedded metal contact located on the two-step deep trench isolation. This increases the vertical distance between the two contacts, thereby suppressing both EBD and DCR. The metal filling in the deep trench functions as an optical shield between pixels and contributes to the suppression of optical crosstalk, which is caused by hot carrier emission through avalanche multiplication [22,23,24]. This two-step trench decreases the total width by integrating two functions of the embedded contact and metal shield into a single trench structure. Additionally, the metal serves as low-impedance metal wiring in the SPAD array, as shown in Figure 1b. This “embedded power grid” reduces the voltage drop across the wiring even for a significant multiplication current in a large SPAD array and under conditions of high illumination.

A polysilicon resistor, *R*_k_, is integrated into the pixel by inserting it in series with the SPAD and quenching circuit, as shown in Figure 1c, and can reduce the amplitude of the voltage swing at the *V*_in_ node, resulting in SPAD multiplication and contributing to CPE reduction. Figure 1d shows a transmission electron microscope (TEM) image of the fabricated 3.06 μm pitch SPAD pixel. The embedded metal contact and the polysilicon resistor on the pixel are successfully integrated. The Si thickness for the SPAD pixel is approximately 2.5 μm.

**Figure 2:**
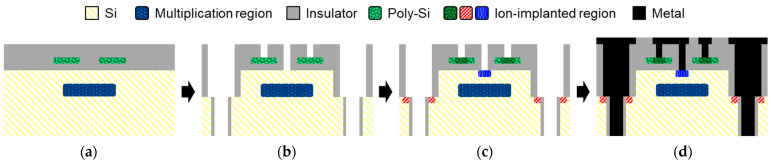
Fabrication process of the SPAD pixel with the embedded metal contact on two-step full trench isolation. (**a**) Ion implantation for the multiplication region and the formation of the poly-Si resistor. (**b**) Etching of the two-step full trench and contact hole. (**c**) Ion implantation for the contact impurities. (**d**) Metal filling and etching.

Figure 2a–d shows a schematic diagram outlining the fabrication process for the embedded metal contact with the poly-Si resistor. The full trench isolation and contact holes are etched after ion implantation for the avalanche region and formation of the poly-Si resistor. After etching, the contact region for cathode contact, anode embedded contact, and poly-Si resistor are implanted with impurities using a self-aligned process. Tungsten metal is filled and etched for the embedded metal and the first wiring layer.

The depth and width of the 1st step of the trench (the wider trench) are important for the design of the SPAD pixel, as this determines the distance between the cathode and anode contact. The width is optimized for the metal filling, which must be sufficient for the optical shield and for the thickness of the side wall oxide, which must be sufficient to relax the electrical field on the pixel surface. The depth is optimized to match the depth of the avalanche region. The self-aligning contact formation process and the two-step trench structure, combining the embedded metal contact with the full trench isolation for the optical shield, help minimize the size of the two-step trench and reduce the EBD.

### 2.2. Pixel Potential Design

**Figure 3:**
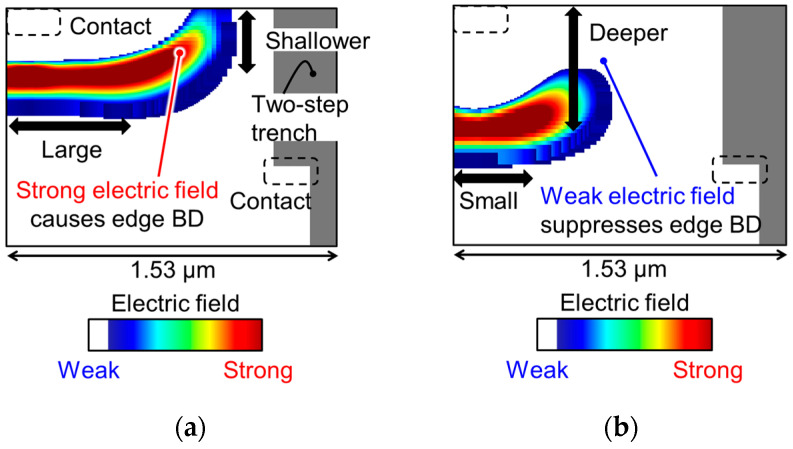
Contour plot of the electric field on the multiplication region estimated by TCAD simulation with (**a**) basic potential design and (**b**) optimized potential design.

Figure 3 shows the distribution of the electric field on the avalanche multiplication region in the pixel, simulated with the pixel structure using technology computer-aided design (TCAD) simulation. Figure 3a shows the electric field based on the same design concept for a SPAD pixel in our previous pixel with 6.12 μm pitch [12] but using an embedded metal contact. The electric field on the pixel surface can be weakened (blue region in Figure 3a) because of the increased vertical distance between the surface cathode contact and embedded anode contact. However, the electric field at a slightly deeper point from the surface is strong, and a certain level of EBD can occur even with the embedded metal contact in this basic design. Figure 3b shows the optimized potential design for weakening the electric field. We reduce the size and increase the depth of the avalanche multiplication region by changing the ion implantation process condition. The size reduction suppresses the spread of the high electric field region, and this region can be moved away from the pixel surface by increasing the depth. Thus, EBD can be suppressed with the optimized design. The deeper multiplication region causes a large variation of the breakdown voltage; however, the multiplication region in the optimized design is still shallower than that in the virtual guard ring design [15] owing to the embedded metal contact; thus, the variation can be smaller than that in [15].

## 3. Results and Discussion

### 3.1. Measurement Results of the Pixel Characteristics

**Figure 4:**
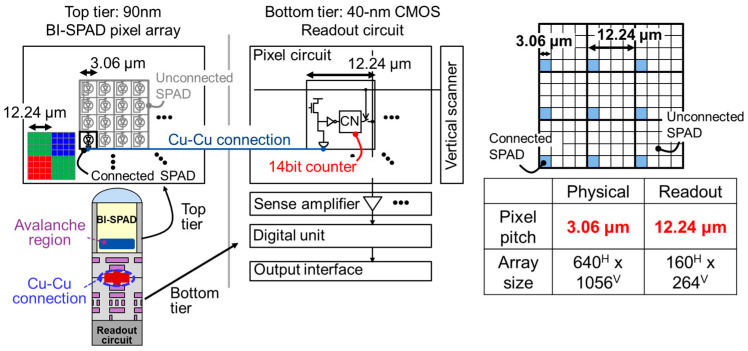
Implementation of a proof-of-concept prototype for the 3.06 μm pitch SPAD pixels. On-chip color filter is placed with 12.24 μm pitch bayer arrangement, depicted in the inset of the top tier pixel array.

Figure 4 shows schematics and specifications of a proof-of-concept prototype for the 3.06 μm pitch SPAD pixels. The back-illuminated 3.06 μm pitch 640 × 1056-pixel array is stacked on a 12.24 μm pitch of 160 × 264 photon-counting circuits array with a 14-bit counter via Cu-Cu connections. The photon-counting circuit pitch is larger than the SPAD pixel pitch due to the large number of in-pixel counter bits. Therefore, only one of the 16 SPAD pixels is connected to the readout circuit.

#### 3.1.1. Breakdown Voltage

**Figure 5:**
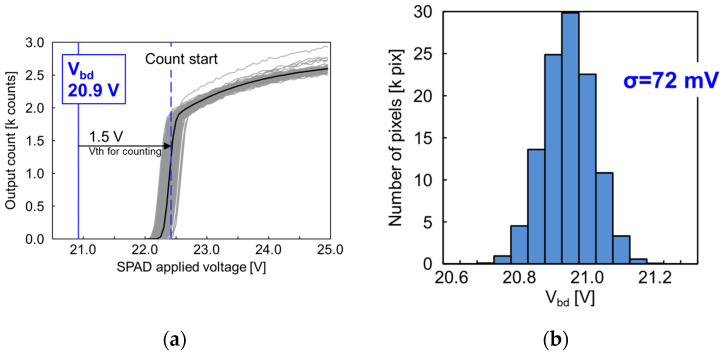
Measurement results of (**a**) SPAD breakdown operation and *V*_bd_. The gray solid lines show the characteristics of each pixel (only 256 extracted pixels) and the black solid line shows the median of all the pixels. The count starting point is approximately 22.4 V (vertical dashed blue line) and the breakdown voltage is 20.9 V (vertical solid blue line), which is less than 1.5 V of threshold voltage from the count starting point. (**b**) The histogram of *V*_bd_ is estimated from the I-V curve of each pixel in (**a**). The standard deviation of the *V*_bd_ is 72 mV. The results were measured at 25 °C.

Figure 5a shows the photon-counting operation with increasing SPAD applied voltage (*V*_dd_-*V*_SPAD_ in Figure 1c) at 25 °C. The counting operation starts at approximately 22.4 V, which shows that the SPAD pixel with a pitch of 3.06 μm pitch works successfully as a photon-counting pixel. The counting start point is determined by the SPAD breakdown voltage and the threshold voltage (*V*_th_) in the output inverter. The SPAD multiplication occurs when the SPAD applied voltage exceeds the breakdown voltage (*V*_bd_) and the inverter input node (*V*_in_) is swung with equal amplitude to the excess bias (*V*_ex_) from the breakdown voltage. The count operation starts with *V*_ex_ exceeding *V*_th_; thus, the counting start point is equal to the sum of *V*_bd_ and *V*_th_. Consequently, the *V*_bd_ of the pixel can be calculated as 20.9 V with 22.4 V of the counting start point minus 1.5 V of the *V*_th_. Figure 5b shows the variation of *V*_bd_ over the entire pixel array. The variation is 72 mV, which is smaller than that in our previous 6.12 μm pitch pixel [12].

**Figure 6:**
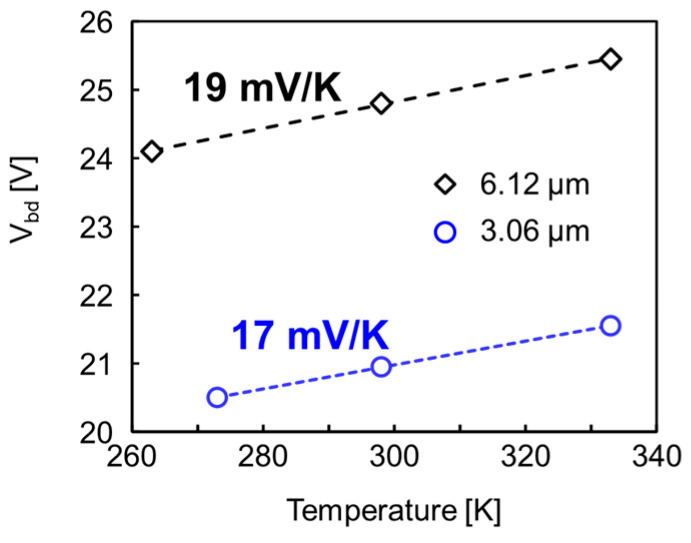
Temperature dependence of *V*_bd_ for the 3.06 μm pitch pixel (blue open circle) and comparison with the values of the previous 6.12 μm pitch pixel [12] (black open diamond). The dashed lines are linear fittings and the values correspond to the slopes of the individual lines.

Figure 6 shows the measured temperature dependence of the breakdown voltage for the pixel array with 3.06 μm pitch. The value of temperature dependence is approximately 17 mV/K and this value is almost the same as our previous pixel with 6.12 μm pitch [12].

These results successfully demonstrate the stable operation of the 3.06 μm pitch pixel array in this work compared to the larger 6.12 μm pitch pixel array in our previous work [12].

#### 3.1.2. Photo Response Non-Uniformity

**Figure 7:**
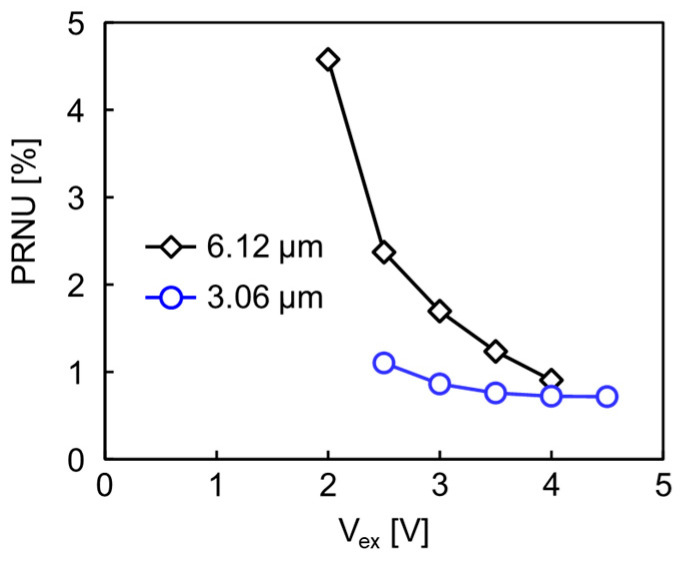
*V*_ex_ dependence of PRNU for the 3.06 μm pitch pixel array (blue open circle and line) in comparison to the previous 6.12 μm pitch pixel array (black open diamond and line). The PRNU of the 3.06 μm pitch pixel array is much smaller than that of the 6.12 μm pitch pixel array [12] with the small *V*_ex_ due to the optimized potential design of the avalanche region.

Figure 7 shows the measured excess bias dependence of the photo response non-uniformity (PRNU) for the 3.06 μm pitch pixel array and compares it with the values of the previous 6.12 μm pitch pixel array [12]. The PRNU is estimated as the standard deviation of the output counts in the array of pixels divided by the mean value of the output counts. The PRNU becomes smaller when the excess bias (*V*_ex_) is increased for both pixel pitches and they are almost the same at 4 V of the *V*_ex_. However, the PRNU for the 3.06 μm pitch pixel is much smaller than that of the 6.12 μm pitch pixel at the small *V*_ex_, e.g., at 2.5 V. The difference reflects the smaller *V*_bd_ variation in the 3.06 μm pitch pixel array. The lower variation is the result of optimizing the potential design for the avalanche region.

#### 3.1.3. Dark Count Rate

**Figure 8:**
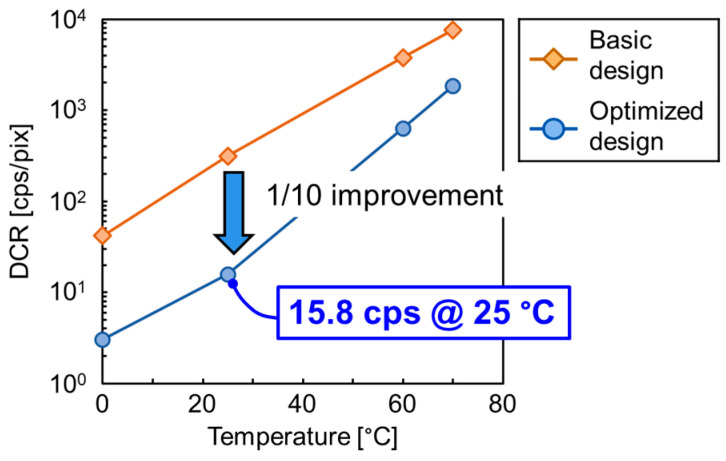
Measurement results for the DCR with *V*_ex_ = 3 V for the two different avalanche potential designs shown in Figure 3.

Figure 8 shows the measurement results of DCR with *V*_ex_ = 3 V for the basic and optimized potential designs from Figure 3. With the optimized design, the DCR at 25 °C is 15.8 cps, while it is 313 cps with the basic design. The DCR in the optimized design is improved by a factor of 10 compared to the basic design. This result shows that the optimized design can successfully reduce the electric field at the pixel edge, as estimated with the potential simulation in Figure 3.

#### 3.1.4. Photo Detection Efficiency

**Figure 9:**
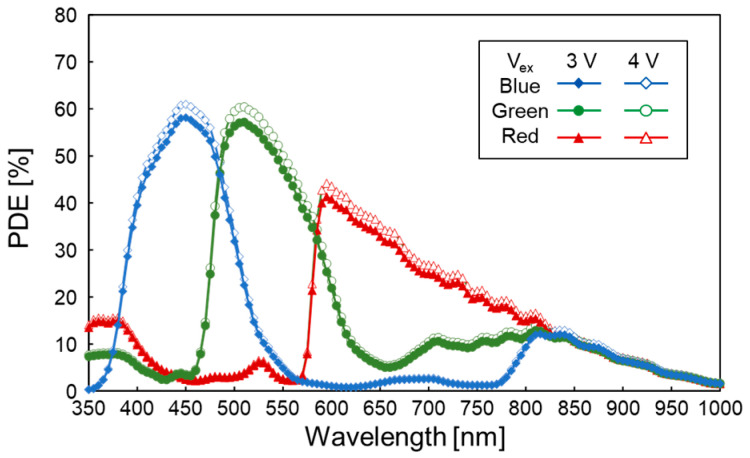
Measurement results of PDE at 25 °C and 3 V (closed markers) and 4 V (open markers) of *V*_ex_. The blue diamond, green circle, and red triangle correspond to pixels with blue, green, and red color filters, respectively.

Figure 9 shows a measured PDE at different wavelengths. The pixel array of the prototype has a Bayer array of on-chip color filters, and the PDE is measured through each color filter. The peak PDE obtained by the green color filter is 57% with *V*_ex_ = 3 V and 60% with *V*_ex_ = 4 V. This can be achieved with a fill factor near 100% owing to the back-illuminated stacked structure and by optimizing the potential slope in the SPAD pixel for electron transfer [24]. The PDE under infrared light, e.g., at a wavelength of 940 nm, is much smaller than the PDE value in previous works [17,18,19]. This is due to the thinner Si thickness (2.5 μm) in our 3.06 μm pitch pixel prototype. If we increase the Si thickness as in the previous works, e.g., to 7 μm, the infrared PDE can be improved.

#### 3.1.5. Crosstalk

**Figure 10:**
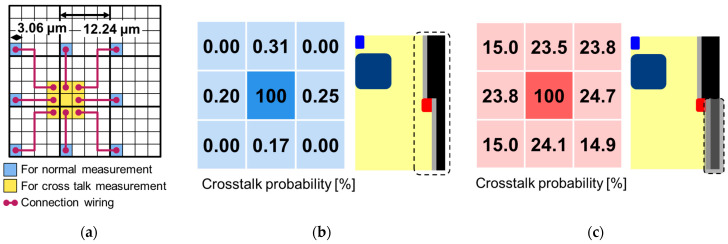
(**a**) Schematic image of special pixel connection which is used for crosstalk measurement where adjacent 3.06 μm pitch 3 × 3 pixels are connected to the 12.24 μm pitch photon counting circuits by metal wiring to read output count with 3.36 μm pitch. Measurement results of crosstalk probability at 25 °C and 3 V of *V*_ex_ (**b**) with and (**c**) without full trench isolation. The matrix corresponds to the position of the measured adjacent 3.06 μm pitch 3 × 3 pixels and the crosstalk probability is calculated from the ratio of the output count of the surrounding eight pixels to the output count of the middle-center pixel. The inset shows the schematic images of the trench structures.

Figure 10 shows the measurement results of crosstalk probability with and without full trench isolation. For the crosstalk measurement, a special pixel connection is used in which adjacent 3.06 μm pitch 3 × 3 pixels are connected to the 12.24 μm pitch photon-counting circuits by metal wiring, as shown in Figure 7a. Figure 7b shows that the crosstalk probability with the two-step full trench isolation is less than 0.4%, while Figure 7c shows that the crosstalk probability without the full trench isolation under the embedded contact is more than 20%. The total crosstalk probabilities with eight surrounding pixels are 0.93% for Figure 7b and 164.8% for Figure 7c. These results highlight the advantage of the two-step full trench isolation, which combines the embedded metal contact with the full trench isolation for the optical shield to prevent crosstalk.

#### 3.1.6. Captured Image

**Figure 11:**
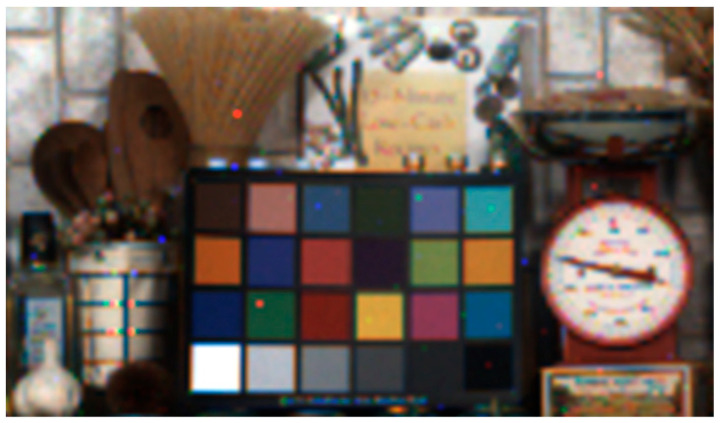
Image captured using the 3.06 μm pitch pixel array and 12.24 μm pitch photon counting circuit. DSNU may cause a few defects.

A color image was successfully captured by using the prototype 3.06 μm pitch SPAD pixel array with an on-chip color filter, as shown in Figure 11. The 160 × 264 pixels color image was obtained with an exposure time of 1/60 s at room temperature. Only a few defects occurred, possibly due to dark signal non-uniformity (DSNU). Improving the DSNU is one of the future challenges for reducing pixel size.

### 3.2. CPE Reduction with Polysilicon Resistor

#### 3.2.1. Theory

**Figure 12:**
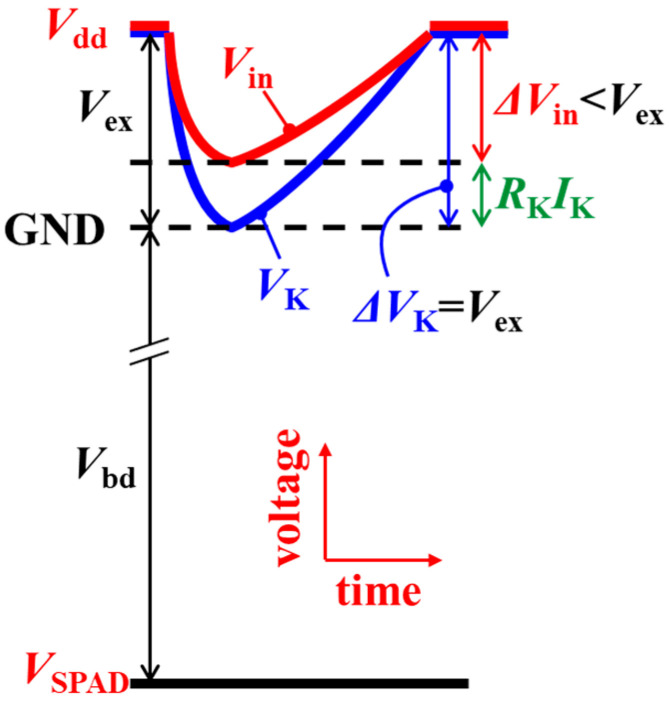
Schematic potential diagram of *V_K_* and *V*_in_ with SPAD multiplication.

The polysilicon resistor on the SPAD pixel can reduce CPE *Q*_CPE_ with SPAD multiplication using the following equation:

(1) $$Q_{\mathrm{CPE}}=C_{K}V_{ex}+C_{in}\left(V_{ex}-R_{K}I_{K}\right)$$

where *C*_K_ is the capacitance at the SPAD cathode before the polysilicon resistor, and *C*_in_ is the total parasitic capacitance after the resistor to the input node of the output inverter. *R*_K_ is the resistance of the polysilicon resistor, and *I*_K_ is the current at the SPAD cathode, as shown in Figure 1c. *C*_in_ is much larger than *C*_K_ because of the Cu-Cu connection and the metal wiring in the CMOS circuits and dominates the CPE. The contribution of *C*_in_ can be reduced with *R*_K_ because the amplitude of the voltage swing at the input node of the inverter (Δ*V*_in_) by SPAD multiplication is reduced by the voltage drop with *R*_K_ and *I*_K_, as shown in Figure 12.

#### 3.2.2. CPE Reduction Measurement Results

**Figure 13:**
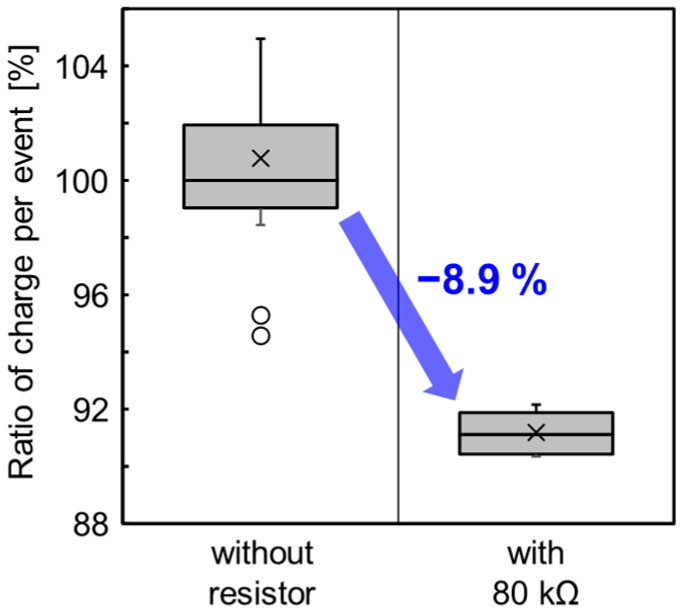
Ratio of CPE with 80 kΩ resistor to those without the resistor. The median of the CPE without resistor is 100%. The symbols × means average value and the circle means outlier from normal distribution.

Figure 13 shows the measurement results of the ratio of the CPE with an 80 kΩ polysilicon resistor to those without the resistor. The CPE is successfully reduced by 8.9% with the resistor. The CPE is estimated based on the measured current at the cathode, the photon counts, and the exposure time.

The CPE reduction is important for high-resolution photon counting with large array sizes. The CPE is an important factor for power consumption in photon counting because the power is proportional to the CPE and the number of counted photons. For a larger array of photon-counting SPAD pixels, the CPE must be reduced to suppress the increase in power consumption. The results of the CPE reduction with a poly-Si resistor show the superiority of our pixel structure for the high-resolution photon-counting image sensor. However, the amplitude reduction of the *V*_in_ voltage swing with the poly-Si resistor increases the apparent *V*_th_ of the count starting voltage. The counting circuit must be carefully designed for the readout of the smaller voltage swing. In addition, the poly-Si resistor reduces the recharge current and increases the deadtime. The resistance of the poly-Si resistor must be optimized with consideration of these adverse effects.

### 3.3. Impedance Reduction of the Embedded Power Grid

#### 3.3.1. The Impedance Estimation

**Figure 14:**
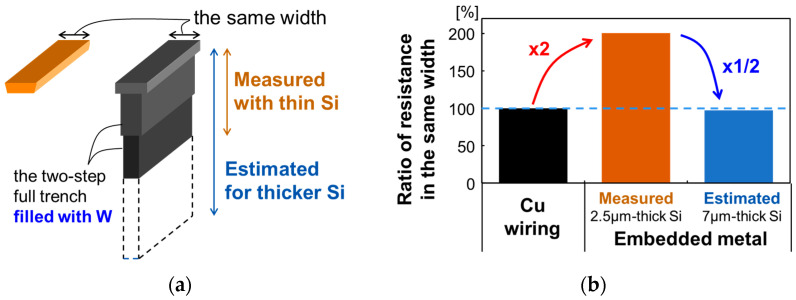
(**a**) Schematic image of the typical copper wiring and the embedded metal wiring (**b**) measurement results of the resistance of the embedded metal wiring compared with typical copper wiring with the same top width and a fabricated 2.5 μm Si thickness and the estimated resistance with 7 μm Si thickness.

In the prototype, we use an embedded power grid for the anode wiring in the pixel array, which replaces the typical copper wiring. Here, we measure the impedance of the embedded power grid and compare it to the impedance of the typical copper wiring. Figure 14a shows the schematic image of the typical copper wiring and the embedded metal wiring in this work. While the copper wiring has a simple inverse tapered shape, the embedded metal has a complicated shape consisting of three parts. The top, widest part is the metal for the wiring on the pixel, and the middle and the bottom narrower parts correspond to the two-step full trench. To compare the resistance between the two different wirings, we assume the top width on the surface is identical. The depth of the embedded metal in the middle part is determined by the etching process of the trench and the depth in the bottom part is determined by the SPAD Si thickness. Figure 14b shows the measurement results of the resistance of the embedded metal wiring compared to typical copper wiring with the same top width and a fabricated 2.5 μm thickness. The measured resistance is twice that of the typical copper wiring. This is because the power grid is filled with tungsten and the resistivity is higher than that of copper. The resistance can be reduced by increasing the Si thickness. The estimated resistance with a 7 μm thick Si decreases to the same level as that of the copper wiring. The contribution of each of the three parts to the total measured resistance is estimated from the volume of each part, which is measured in Figure 1d, and the resistance with the 7 μm thick Si is estimated from the contribution. This shows that the embedded metal wiring in addition to the copper wiring can decrease the resistance of the power supply.

#### 3.3.2. Photo Response Shading Measurement Results

**Figure 15:**
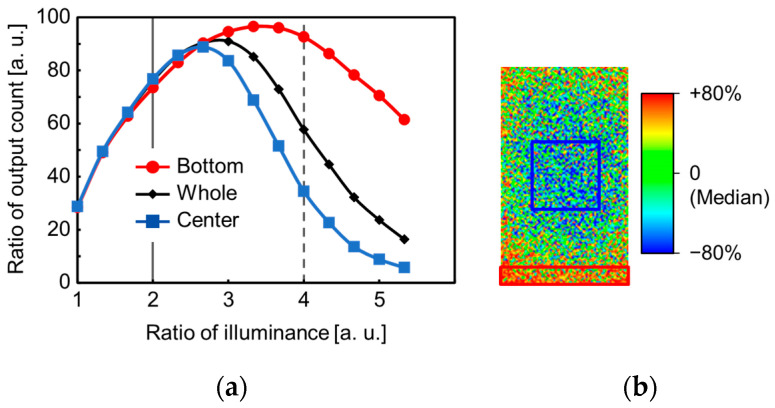
(**a**) The measurement results of the median of the output count as a function of the illuminance in the whole area (black diamond and line), the bottom area (red circle and line), and the center area (blue square and line) in the SPAD pixel array are plotted, respectively. (**b**) The heat map of relative output count in the pixel array at the illuminance of the dashed gray line in Figure 15a. The red square and blue square correspond to the area for median calculation for the bottom area and center area, respectively, in (**a**). The heat map calculated from the output count and its median value for the whole area of the pixel array.

Figure 15a shows the measurement results of the output count as a function of the illuminance, while Figure 15b shows the heat map of the relative output count in the pixel array at the illuminance of the dashed gray line in Figure 15a. In Figure 15a, the median of the output counts in the complete area, the bottom area, and the middle-center area of the SPAD pixel array are plotted. The difference between the medians of the bottom and center area indicates photo response shading in the array as a result of voltage drop caused by SPAD multiplication current. This plot shows the photo response curve under extremely high illuminance. The photo response is saturated under such high illuminance because the photon incidence frequency is higher than the deadtime of a SPAD pixel and a pile-up of the avalanche multiplication occurs. While the measured medians of the output count in the bottom and middle-center area are almost the same at lower illuminance (on the vertical solid line in Figure 15a), the output count in the middle-center area is much smaller than that in the bottom area at higher illuminance (on the vertical dashed line in Figure 15a). This is because the middle-center photo-response peak has a lower illuminance level.

Based on these results, we can conclude that the main voltage drop occurs in the cathode wiring, while the anode voltage drop does not occur due to the sufficiently low resistance of the embedded metal wiring. If the voltage drop occurs on the anode wiring, the PDE is lower because the actual excess bias is smaller, and the slope of the photo response in the center of the array is reduced. However, the results indicate that the illuminance level at the peak of the photo response is lower in the middle-center of the array and the photo response decreases more rapidly with high illuminance. This indicates the deadtime shading in the array due to the cathode voltage drop. If the cathode voltage drops, the recharge current decreases, and the deadtime increases. The longer deadtime causes the pile up even at the lower illuminance level. The deadtime shading can be clearly seen in Figure 15b. The output count in the center of the pixel array is smaller than that at the edge of the pixel array.

These results show that the embedded power grid is effective for powering the anode in the high-resolution photon-counting image sensor. In the high-resolution photon-counting image sensor, the multiplication current increases due to the large array size, and the suppression of the voltage drop by a low-impedance power supply wiring is mandatory. The voltage drop in the cathode wiring must also be reduced. In the future, we will optimize the wiring design.

## 4. Conclusions

**Figure 16:**
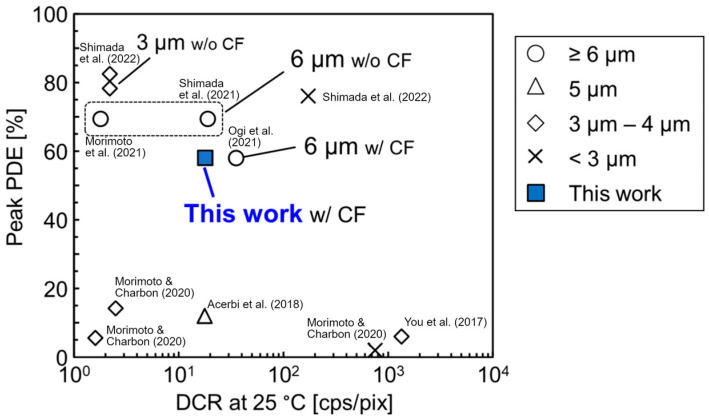
Comparison of PDE and DCR with previous research. The largest value in the manuscript is used for [14] because the manuscript lacks data for the peak PDE; Ogi et al. (2021) [12]; Acerbi et al. (2018) [14]; You et al. (2017) [15]; Morimoto & Charbon (2020) [16]; Shimada et al. (2021) [17]; Morimoto et al. (2021) [18]; Shimada et al. (2022) [19].

Figure 16 presents the DCRs and PDEs of previous studies and the present study. Here, the PDE obtained in this study is not the highest value, but it is significantly higher than that of previous research [14,15,16], which uses 5 μm pitch or smaller pixels, while the DCR is comparable to that of previous research using 6 μm pitch pixels [12,17,18]. The best PDE and DCR with the smallest pixel pitch (3.3 or 3 μm) were reported in [19], which were better than those of the present study. However, comparing the other pixel characteristics, as shown in Table 2, revealed that the crosstalk is lower than those of [19]. This is because of the full trench isolation with embedded metal and the extension of the metal over the silicon surface, as shown in Figure 1a,d. In addition, the integration of a power grid and the polysilicon resistor on SPAD pixels can contribute to a reduced voltage drop in anode power supply and reduced power consumption with SPAD multiplication, respectively, in a large SPAD pixel array for a high-resolution photon-counting image sensor.

## Figures and Tables

**Table 1:** Comparison between proposed SPAD pixel designs/structures for shrinking pixel size.

	[16] Well Sharing (Conventional)	[15] Virtual Guard Ring	[16] Guard-Ring Sharing	This WorkEmbedded Contact
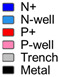	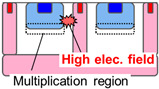	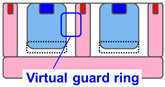	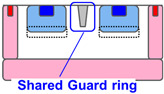	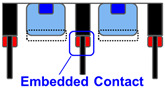
Electric field at pixel edge	High	Low	Low	Low
Vbd variation	Small	Large	Small	Small
Well impedance	Low	Low	High	Low
Fabrication	Simple	Simple	Complex	Highly complex

**Table 2:** Comparison between the pixel characteristics of this study and those of previous research.

	Unit	[12]	[17]	[18]	[14]	[15]	[16]			[19]			This Research
Pixel pitch	μm	6.12	6	6.39	5	3	4	3	2.2	3.3	3.0	2.5	3.06
Array size	-	160 × 264	N/D	2072 × 1548	N/D	4 × 4	4 × 4			N/D			160 × 264
Techno-logy	-	BI-3D 90 nm	BI-3D 90 nm	BI-3D 90 nm	FI	FI 130 nm	FI 180 nm			BI-3D 90 nm			BI-3D 90 nm
V_bd_	V	N/D	22	30	N/D	15.8	22.1	23.6	32.35	19	19	18	20.9
V_ex_	V	3	3	2.5	5.8 *^3^	1.2	6	6	6	3	3	3	3
Peak PDE	%	58	69.4 *^1^	69.4	12 *^2^*^3^	6 *^3^	14.2	5.6	2	82.5	78.3	76.1	57
DCR @25 °C	cps	35.4	19	1.8	17.3 *^3^	1343	2.5	1.6	751	2.2	2.2	173	15.8
Cross- talk	%	N/D	0.5	N/D	4.9 *^3^	<0.2 *^4^	3.57	2.75	2.97	0.85	0.95	1.0	<0.4

*^1^ Referred from [19]. *^2^ No data for peak PDE. The largest value in the referenced article is used. *^3^ No numerical value is expressed in the referenced articles. The author calculated the value from the graphs in the referenced articles. *^4^ *V*_ex_ = 1 V.

## Data Availability

The data presented in this study are available on request from the corresponding author. The data are not publicly available due to the confidentiality of the corporate activity.

## References

[1] Ximenes A.R., Padmanabhan P., Lee M.J., Yamashita Y., Yaung D.N., Charbon E. A 256×256 45/65nm 3D-Stacked SPAD-Based Direct TOF Image Sensor for LiDAR Applications with Optical Polar Modulation for up to 18.6dB Interference Suppression. Proceedings of the 2018 IEEE International Conference on Solid-State Circuits (ISSCC).

[2] Henderson R.K., Johnston N., Hutchings S.W., Gyongy I., Abbas T.A., Dutton N., Tyler M., Chan S., Leach J. A 256×256 40nm/90nm CMOS 3D-Stacked 120dB Dynamic-Range Reconfigurable Time-Resolved SPAD Imager. Proceedings of the 2019 IEEE International Conference on Solid-State Circuits (ISSCC).

[3] Kumagai O., Ohmachi J., Matsumura M., Yagi S., Tayu K., Amagawa K., Matsukawa T., Ozawa O., Hirono D., Shinozuka Y. A 189×600 Back-Illuminated Stacked SPAD Direct Time-of-Flight Depth Sensor for Automotive LiDAR System. Proceedings of the 2021 IEEE International Conference on Solid-State Circuits (ISSCC).

[4] Manuzzato E., Tontini A., Seljak A., Perenzoni M. A 64 64-Pixel Flash LiDAR SPAD Imager with Distributed Pixel-to-Pixel Correlation for Background Rejection, Tunable Automatic Pixel Sensitivity and First-Last Event Detection Strategies for Space Applications. Proceedings of the 2022 IEEE International Conference on Solid-State Circuits (ISSCC).

[5] Yin C., Yeh S.F., Huang C.Y., Tu H.Y., Wu M.H., Wang T.J., Huang K.C., Chao C.Y.P. A 320×232 LiDAR Sensor with 24dB TimeAmplified and Phase-Revolved TDC. Proceedings of the 2023 International Image Sensor Workshop Edinburg.

[6] Ma J., Hondongwa D., Fossum E.R. Jot devices and the quanta image sensor. Proceedings of the 2014 IEEE Electron Devices Meeting (IEDM).

[7] Ingle A., Velten A., Gupta M. High flux passive imaging with single-photon sensors. Proceedings of the IEEE/CVF Conference on Computer Visual Pattern Recognition (CVPR).

[8] Dutton N.A.W., Gyongy I., Parmesan L., Gnecchi S., Calder N., Rae B.R., Pellegrini S., Grant L.A., Henderson R.K. (2016). A SPAD-based QVGA image sensor for single-photon counting and quanta imaging. IEEE Trans. Electron Devices.

[9] Perenzoni M., Massari M., Perenzoni D., Gasparini L., Stoppa D. (2016). A 160×120 pixel analog-counting single-photon imager with time-gating and self-referenced column-parallel A/D conversion for fluorescence lifetime imaging. IEEE J. Solid-State Circuit.

[10] Abbas T.A., Dutton N.A.W., Almer O., Pellegrini S., Henrion Y., Henderson R.K. Backside illuminated SPAD image sensor with 7.83μm pitch in 3D-stacked CMOS technology. Proceedings of the 2016 IEEE Electron Device Meeting (IEDM).

[11] Hutchings S.W., Johnston N., Gyongy I., Abbas T.A., Dutton N.A.W., Tyler M., Chan S., Leach J., Henderson R.K. (2019). A Reconfigurable 3-D-Stacked SPAD Imager With In-Pixel Histogramming for Flash LIDAR or High-Speed Time-of-Flight Imaging. IEEE J. Solid-State Circuits.

[12] Ogi J., Takatsuka T., Hizu K., Inaoka Y., Zhu H., Tochigi Y., Tashiro Y., Sano F., Murakawa Y., Nakamura M. (2021). A 124-dB Dynamic-Range SPAD Photon-Counting Image Sensor Using Subframe Sampling and Extrapolating Photon Count. IEEE J. Solid-State Circuits.

[13] Ota Y., Morimoto K., Sasago T., Shinohara M., Kuroda Y., Endo W., Maehashi Y., Maekawa S., Tsuchiya H., Abdelghafar A. A 0.37W 143dB-Dynamic-Range 1Mpixel Backside-Illuminated Charge-Focusing SPAD Image Sensor with Pixel-Wise Exposure Control and Adaptive Clocked Recharging. Proceedings of the 2022 IEEE International Conference on Solid-State Circuits (ISSCC).

[14] Acerbi F., Gola A., Regazzoni V., Paternoster G., Borghi G., Zorzi N., Piemonte C. (2018). High Efficiency, Ultra-High-Density Silicon Photomultipliers. IEEE J. Top. Quantum Elec..

[15] You Z., Parmesan L., Pellegrini S., Henderson R.K. 3μm Pitch, 1μm Active Diameter SPAD Arrays in 130nm CMOS Imaging Technology. Proceedings of the 2017 International Image Sensor Workshop.

[16] Morimoto K., Charbon E. (2020). High fill-factor miniaturized SPAD arrays with guard-ring-sharing technique. Opt. Express.

[17] Shimada S., Otake Y., Yoshida S., Endo S., Nakamura R., Tsugawa H., Ogita T., Ogasahara T., Yokochi K., Inoue Y. A Back Illuminated 6 µm SPAD Pixel Array with High PDE and Timing Jitter Performance. Proceedings of the 2021 IEEE Electron Device Meeting (IEDM).

[18] Morimoto K., Iwata J., Shinohara M., Sekine H., Abdelghafar A., Tsuchiya H., Kuroda Y., Tojima K., Endo W., Maehashi Y. 3.2 Megapixel 3D-Stacked Charge Focusing SPAD for Low-Light Imaging and Depth Sensing. Proceedings of the 2021 IEEE Electron Device Meeting (IEDM).

[19] Shimada S., Otake Y., Yoshida S., Jibiki Y., Fujii M., Endo S., Nakamura R., Tsugawa H., Fujisaki Y., Yokochi K. A SPAD Depth Sensor Robust Against Ambient Light: The Importance of Pixel Scaling and Demonstration of a 2.5μm Pixel with 21.8% PDE at 940nm. Proceedings of the 2022 IEEE Electron Device Meeting (IEDM).

[20] Ogi J., Sano F., Nakata T., Matsumura Y., Kubo Y., Onishi W., Koswaththaghe C.J., Mochizuki T., Tashiro Nakazawa K., Koga F. A Challenge for 3μm SPAD pixel Using Embedded Metal Contact on Deep Trench Pixel Isolation. Proceedings of the International SPAD Sensor Workshop.

[21] Ogi J., Sano F., Nakata T., Kubo Y., Onishi W., Koswaththaghe C.J., Mochizuki T., Tashiro Y., Hizu K., Takatsuka T. A 3.06 μm SPAD Pixel with Embedded Metal Contact and Power Grid on Deep Trench Pixel Isolation for High-resolution Photon-counting. Proceedings of the 2023 International Image Sensor Workshop.

[22] Rech I., Ingargiola A., Spinelli R., Labanca I., Marangoni S., Ghioni M. (2008). A New Approach to Optical Crosstalk Modeling in Single-Photon Avalanche Diodes. IEEE Photonics Technol. Lett..

[23] Kindt W.J., van Zeijl H.W., Middelhoek S. Optical Cross Talk in Geiger Mode Avalanche Photodiode Arrays: Modeling, Prevention and Measurement. Proceedings of the 28th European Solid-State Device Research Conference.

[24] Ito K., Otake Y., Kitano Y., Matsumoto A., Yamamoto J., Ogasahara T., Hiyama H., Naito R., Takeuchi K., Tada T. A Back Illuminated 10μm SPAD Pixel Array Comprising Full Trench Isolation and Cu-Cu Bonding with Over 14% PDE at 940nm. Proceedings of the 2020 IEEE Electron Device Meeting (IEDM).

